# Association of the FRACTURE Index With Bone Mineral Density and Fracture Risk Stratification in Postmenopausal Women: A Cross-Sectional Study

**DOI:** 10.7759/cureus.101267

**Published:** 2026-01-10

**Authors:** Khatri Om Prakash, Raghuveer Chaudhary, Govind Singh

**Affiliations:** 1 Medical and Health, Government District Nahata Hospital, Balotra, IND; 2 Physiology, Dr. S.N. Medical College, Jodhpur, IND; 3 Physical Medicine and Rehabilitation, Dr. S.N. Medical College, Jodhpur, IND

**Keywords:** bone mineral density, dexa, fracture risk, osteoporosis, post menopausal

## Abstract

Introduction

Osteoporosis is a major public health concern among postmenopausal women due to accelerated bone loss and increased susceptibility to fragility fractures. Although bone mineral density (BMD) assessment using dual-energy X-ray absorptiometry (DEXA) is the standard diagnostic tool, fracture risk is influenced by multiple clinical factors beyond BMD alone. The FRACTURE Index is a validated clinical risk assessment tool that integrates several key predictors to estimate future fracture risk.

Objective

To evaluate the association between the FRACTURE Index and bone mineral density and to assess its role in fracture risk stratification among postmenopausal women.

Methods

This hospital-based cross-sectional study included 102 postmenopausal women aged ≥45 years who underwent DEXA scanning. Bone mineral density was classified according to World Health Organization criteria. Future fracture risk was assessed using the FRACTURE Index. Descriptive statistics were used for data summarization, while associations were analyzed using chi-square tests and Pearson correlation coefficients.

Results

Among the participants, 31 women (30.4%) were osteoporotic, 47 (46.1%) were osteopenic, and 24 (23.5%) had normal BMD. A FRACTURE Index score ≥6, indicating high future fracture risk, was observed in 42 women (41.2%). Higher FRACTURE Index scores were significantly associated with lower lumbar spine T-scores (r = −0.759, p < 0.001), increasing age (r = 0.63, p < 0.001), prior fracture history (χ² = 38.07, p < 0.001), and maternal history of fracture (χ² = 25.83, p < 0.001).

Conclusion

The FRACTURE Index effectively identifies postmenopausal women at high risk of future osteoporotic fractures and demonstrates a strong association with bone mineral density. Its integration into routine clinical screening may enhance early risk stratification and preventive strategies, particularly in resource-limited settings.

## Introduction

Osteoporosis is a systemic skeletal disorder characterized by reduced bone strength resulting from diminished bone mass and deterioration of bone microarchitecture, ultimately increasing fracture susceptibility [1]. The condition remains largely asymptomatic until a fracture occurs, leading to significant morbidity, mortality, and healthcare burden [2]. Women are commonly affected, particularly after menopause, due to estrogen deficiency that accelerates bone resorption [3].

Postmenopausal bone loss is most rapid during the early years after menopause, contributing to a substantial decline in bone mineral density (BMD) and an increased risk of fragility fractures [4]. The World Health Organization defines osteoporosis as a BMD T-score ≤ −2.5 measured by dual-energy X-ray absorptiometry (DEXA), while osteopenia is defined by T-scores between −1.0 and −2.5 [1]. While BMD is a strong predictor of fracture risk, it does not fully capture all clinical risk factors contributing to fractures [5].

Epidemiological evidence suggests that a considerable proportion of osteoporotic fractures occur in individuals with osteopenia rather than established osteoporosis, highlighting the limitations of relying solely on BMD measurements [6,7]. Consequently, several clinical risk assessment tools have been developed to improve fracture risk prediction by incorporating non-BMD factors [8].

The FRACTURE Index, developed by the Study of Osteoporotic Fractures Research Group, combines age, previous fracture history, maternal history of hip fracture, body weight, smoking status, mobility, and bone mineral density to estimate future fracture risk [9]. In India, the burden of osteoporosis is rising due to increasing life expectancy, nutritional deficiencies, vitamin D insufficiency, sedentary lifestyle, and limited awareness [10-12]. Early identification of women at high fracture risk is essential to implement timely preventive and therapeutic interventions. This study aimed to evaluate the association between the FRACTURE Index and bone mineral density and to assess its role in fracture risk stratification among postmenopausal women.

## Materials and methods

This hospital-based cross-sectional observational study was conducted at a tertiary care hospital attached to Dr. Sampurnanand (SN) Medical College, Jodhpur, Rajasthan, India, from September 2019 to August 2020. A total of 102 postmenopausal women aged 45 years and above who presented for routine bone mineral density assessment were enrolled. Postmenopausal status was defined as the absence of menstruation for at least 12 consecutive months.

Women with chronic systemic illnesses affecting bone metabolism, secondary causes of osteoporosis, long-term corticosteroid or anti-osteoporotic drug use, or severe skeletal deformities were excluded from the study. All eligible participants provided written informed consent before enrollment. The study was started after approval by the Institutional Ethics Committee (approval number SNMC/IEC/2019/518-520).

Data collection was performed using a structured proforma to record demographic details, clinical history, previous fractures after the age of 50 years, and maternal history of fracture. Bone mineral density was assessed using dual-energy X-ray absorptiometry (DEXA) of the lumbar spine (L1-L4). BMD results were categorized as normal (T-score > −1.0), osteopenia (T-score −1.0 to −2.5), or osteoporosis (T-score ≤ −2.5) in accordance with World Health Organization criteria [1].

Future fracture risk was evaluated using the FRACTURE Index, a previously validated clinical risk assessment tool adapted from the Study of Osteoporotic Fractures [9]. A cumulative FRACTURE Index score was calculated for each participant, with a score of ≥6 considered indicative of high future fracture risk. 

Statistical analysis was performed using SPSS software v.26 (IBM Inc., Armonk, NY, USA). Continuous variables were expressed as mean ± standard deviation, while categorical variables were summarized as number (percentage). Associations between categorical variables were analyzed using chi-square tests, and correlations between continuous variables were assessed using Pearson correlation coefficients. A p-value < 0.05 was considered statistically significant.

## Results

Baseline characteristics

The mean age of the study population was 58.46 ± 9.31 years, and the mean body mass index was 25.29 ± 4.48 kg/m². The mean lumbar spine T-score was −1.90 ± 1.41. Previous fractures after the age of 50 years were reported by 28 women (27.5%), and maternal history of fracture was reported by 27 women (26.5%) (Table 1).

**Table 1:** Baseline Demographic and Clinical Characteristics (n = 102)

Variable	Value
Age (years), mean ± SD	58.46 ± 9.31
Age group, n (%)	
45–54 years	39 (38.2)
55–64 years	36 (35.3)
≥65 years	27 (26.5)
Body mass index (kg/m²), mean ± SD	25.29 ± 4.48
Lumbar spine T-score, mean ± SD	−1.90 ± 1.41
Previous fracture after age 50, n (%)	28 (27.5)
Maternal history of fracture, n (%)	27 (26.5)

Prevalence of low bone mineral density

Based on WHO criteria, osteoporosis was identified in 31 women (30.4%), osteopenia in 47 women (46.1%), and normal BMD in 24 women (23.5%). Increasing age was significantly associated with declining bone mineral density (Table 2).

**Table 2:** Association of Bone Mineral Density Categories with Age Group

Age Group (years)	Normal n (%)	Osteopenia n (%)	Osteoporosis n (%)	Total n (%)	χ² value	p-value
45–54	16 (41.0)	19 (48.7)	4 (10.3)	39 (100)	14.62	0.006
55–64	6 (16.7)	18 (50.0)	12 (33.3)	36 (100)		
≥65	2 (7.4)	10 (37.0)	15 (55.6)	27 (100)		
Total	24 (23.5)	47 (46.1)	31 (30.4)	102 (100)		

Association with fracture history

A history of fracture after 50 years of age showed a strong association with osteoporosis (χ² = 38.07, p < 0.001). Similarly, maternal history of fracture was significantly associated with lower bone mineral density categories (χ² = 25.83, p < 0.001).

Distribution of FRACTURE Index scores

FRACTURE Index scores ranged from 0 to 13, with a mean score of 3.94 ± 3.04. High future fracture risk (score ≥6) was observed in 42 women (41.2%). Higher FRACTURE Index scores were predominantly observed among women with osteopenia and osteoporosis (Table 3).

**Table 3:** Distribution of FRACTURE Index Scores

FRACTURE Index Score	Normal n (%)	Osteopenia n (%)	Osteoporosis n (%)	Total n (%)	χ² value	p-value
0–2	24 (100)	0 (0)	0 (0)	24 (100)	78.94	<0.001
3–4	0 (0)	27 (100)	0 (0)	27 (100)		
5	0 (0)	9 (100)	0 (0)	9 (100)		
6–7	0 (0)	11 (61.1)	7 (38.9)	18 (100)		
8–13	0 (0)	0 (0)	24 (100)	24 (100)		

Correlation analysis

A strong inverse correlation was observed between FRACTURE Index score and lumbar spine T-score (r = −0.759, p < 0.001). Age demonstrated a significant positive correlation with FRACTURE Index score (r = 0.63, p < 0.001) (Table 4).

**Table 4:** Correlation of FRACTURE Index Score with Age and Bone Mineral Density

Variable Compared	Correlation coefficient (r)	p-value
FRACTURE Index score vs. Age	0.63	<0.001
FRACTURE Index score vs. Lumbar spine T-score	−0.759	<0.001

## Discussion

The present study demonstrates a significant association between the FRACTURE Index and bone mineral density categories, with higher FRACTURE Index scores corresponding to lower bone mineral density and greater prevalence of osteoporosis, supporting its role as a clinical risk stratification tool rather than a direct predictor of future fracture outcomes.

The Fracture Risk Assessment Tool (FRAX), developed by the World Health Organization Collaborating Centre for Metabolic Bone Diseases, University of Sheffield, Sheffield, United Kingdom, is currently the most widely used fracture risk assessment tool internationally and estimates the 10-year probability of major osteoporotic and hip fractures using multiple clinical risk factors with or without femoral neck bone mineral density [13].** **In contrast, the FRACTURE Index provides a simpler and more easily applicable approach to fracture risk stratification.

The prevalence of osteoporosis observed in the present study (30.4%) is comparable to findings from Indian studies conducted by Mithal et al. and Marwaha et al., which reported osteoporosis prevalence ranging between 25% and 35% among postmenopausal women [10,12]. This alignment suggests that the burden of osteoporosis in Indian women remains substantial and consistent across different regions. Challenges in osteoporosis management in India, including limited screening and treatment gaps, have also been highlighted in previous reviews [14]. Similar prevalence rates have also been reported in international cohorts, supporting the global relevance of these findings [15].

Advancing age was significantly associated with declining bone mineral density in this study, a finding that aligns with established evidence demonstrating accelerated bone loss following menopause due to estrogen deficiency [4,15]. The positive correlation between age and FRACTURE Index score further supports the role of age as a major determinant of fracture risk, consistent with observations from the Study of Osteoporotic Fractures and other international fracture risk models [9,16].

A key finding of this study was the strong association between previous fracture history and osteoporosis. This observation is in agreement with meta-analyses and longitudinal studies showing that a prior fragility fracture is one of the strongest predictors of subsequent fractures, independent of bone mineral density [6,15]. Similarly, the significant association between maternal history of fracture and low bone mineral density observed in the present study aligns with genetic studies highlighting hereditary contributions to skeletal fragility [17].

Importantly, a substantial proportion of women classified as high risk by the FRACTURE Index were osteopenic rather than osteoporotic. This finding is consistent with reports by Siris et al. and Bliuc et al., which demonstrated that many fragility fractures occur in individuals with osteopenia rather than established osteoporosis [6,7]. These results expand upon existing literature by reinforcing the limitation of relying solely on BMD for fracture risk assessment and underscore the added value of clinical risk tools such as the FRACTURE Index.

The strong inverse correlation between FRACTURE Index scores and lumbar spine T-scores observed in this study further validates the clinical utility of the FRACTURE Index. Similar correlations have been reported in studies comparing fracture risk assessment tools with BMD measurements, suggesting that integrated approaches improve risk stratification accuracy [18-20]. In the Indian context, where access to advanced diagnostic facilities may be limited, the FRACTURE Index offers a practical and cost-effective adjunct for identifying high-risk individuals.

Overall, the findings of this study align with existing Indian and international literature while expanding current knowledge by demonstrating the applicability of the FRACTURE Index in an Indian postmenopausal population. The results support the integration of clinical risk assessment tools into routine osteoporosis screening programs to improve early identification and prevention of fractures.

Limitations

The cross-sectional design precludes causal inference and direct prediction of future fracture events. The absence of longitudinal fracture outcomes limits the assessment of predictive validity. The single-center setting and modest sample size may affect generalizability.

## Conclusions

The FRACTURE Index is significantly associated with bone mineral density and effectively categorizes postmenopausal women into fracture risk groups. While it does not allow prediction of future fracture events in the absence of longitudinal follow-up, it may serve as a practical clinical tool for fracture risk stratification in routine clinical practice. Early identification of high-risk individuals can facilitate timely preventive interventions, reduce fracture-related morbidity, and improve long-term musculoskeletal health outcomes. Wider incorporation of such integrated risk assessment approaches into routine clinical practice may significantly reduce the public health burden of osteoporosis.
